# TRACE: applying AI language models to extract ancestry information from curated biomedical literature

**DOI:** 10.3389/fdgth.2025.1608370

**Published:** 2025-09-19

**Authors:** Alison M. Veintimilla, Chintan K. Acharya, Connie J. Mulligan, Ruogu Fang, Erika Moore

**Affiliations:** ^1^ Fischell Department of Bioengineering, University of Maryland, College Park, MD, United States; ^2^ Department of Computer and Information Science, University of Florida, Gainesville, FL, United States; ^3^ Department of Anthropology, Genetics Institute, University of Florida, Gainesville, FL, United States; ^4^ J. Crayton Pruitt Family Department of Biomedical Engineering, University of Florida, Gainesville, FL, United States

**Keywords:** ancestry representation, automated text mining, cell line identification, biomedical research equity, AI language models, open source tool

## Abstract

**Introduction:**

Ancestry reporting is essential to ensure transparency and proper representation in biomedical studies. However, manually extracting this information from study texts is time-consuming and inefficient. In this paper, we present TRACE (Tool for Researching Ancestry and Cell Extraction), powered by GPT-4 and web-crawling, to automate ancestry identification by detecting cell lines or cultures in texts and tracing their ancestry.

**Methods:**

TRACE extracts cell lines and primary cultures from research articles and follows web sources to determine their ancestry. We compared TRACE's outputs to a manually generated database to confirm its performance in identifying and verifying ancestry information.

**Results:**

The results reveal an overrepresentation of European/White samples and significant underreporting. TRACE enables large-scale, systematic ancestry analysis—a valuable resource for researchers and agencies assessing biases in sample selection.

**Conclusions:**

As an open-source tool, TRACE it facilitates broader use to evaluate and improve ancestry representation in biomedical research.

## 1 Introduction

Researchers within the field of genetics have long understood that combined social/environmental and genetic factors have profound effects on disease expression and outcome (1, 2). The factor of ancestry can impact both health outcomes and therapeutic interventions. Meta-analysis amongst different disciplines within biology have begun to interrogate the distribution of ancestral representation within their field's given landscape. Additionally, national agencies such as the National Academies of Sciences, Engineering and Medicine (NASEM) have employed task forces to evaluate the use of ancestral descriptors in biomedical research. Many meta-analyses have concluded that there is an overwhelming representation of White/European individuals within genetic and disease studies (3–5). These studies, while incredibly helpful in pointing towards deficits within fields, are oftentimes time-consuming and require a large degree of labor in order to manually extract ancestry-related information. Herein we describe the establishment of a machine learning model, *Tool for Researching Ancestry and Cell Knowledge* (TRACE), to both expedite and also increase the scalability of such analysis.

Genetic and sociocultural determinants of ancestry have been found to affect health outcomes, cellular engineering efforts, and therapeutic interventions. Previous *in vitro* studies have demonstrated the impact of ancestral backgrounds on biological outputs (6). Differing response expression quantitative trait loci (reQTLs) between European and African-descended primary monocytes were responsible for a significant variation in their respective immune responses (7, 8). Specifically, they possessed genetic variants that caused NCF2 and CCR1 gene downregulation in response to immune signals, mutations that have been linked to systemic lupus erythematosus and hepatocellular carcinoma onset, respectively (7, 9, 10). An innate immune cell-type, macrophages, also exhibited a marked difference in immune response between European and African descent, with a large portion of their gene expression differences at least partly attributed to ancestry (8). In particular, African-descended macrophages displayed a much stronger pro-inflammatory immune response compared to their European counterparts, thought to be partly a result of local adaptation (8). These ancestry-related differences in immune response and gene expression invite further interest into how modern biomedical researchers consider ancestry and genetic background when selecting cell lines for biomedical research.

The development of health inequities amongst different ancestral populations through the stacked effects of both sociocultural and molecular factors can be exemplified through autoimmune disorders, such as systemic lupus erythematosus. This form of lupus disproportionately impacts Hispanic, African American, and Asian populations in the US with a 2–3× greater likelihood of occurrence over White/European counterparts (11). Lupus particularly impacts African American women, with 1 in 250 African American women developing lupus within their lifetime (12). African American women are also more likely to have a severe endophenotype (11, 13, 14). Lupus expression amongst this demographic is exacerbated through social determinants. A recent multivariable linear regression analysis conducted in 2019 found that increased incidences of racial discrimination were associated with African American women having greater lupus activity as well as organ damage (14). These inequities are not limited by genetics but are impacted multi-factorially by other derivatives of ancestral background, social inequity, class, and healthcare access (15). Studies looking at ancestral distribution of samples have advocated for increasing the inclusion of diverse ancestral groups in research and have stressed that without such efforts, precision medicine and personalized healthcare will remain limited and inequitable (3).

With these considerations in mind, biology fields have begun to acknowledge the impact that ancestry plays in the outcomes of *in vitro* studies. Recently, there has been a call to report and consider the ancestry of human-derived primary cells and cell lines within bioengineering platforms (16). A study conducted in 2021 found that most cell lines used in benchtop regenerative engineering studies for 10 major journals were of White/European ancestry, while most primary cells utilized had no ancestry reported. The consensus of this study was that ancestry within these journals was severely underreported (17). A commentary analysis written by Popejoy et al., highlights the significant lack of ancestral diversity in genomic research. The authors reveal that over 80% of participants in genome-wide association studies (GWAS) are of European descent, leading to biased conclusions that limit the generalizability of genetic findings to other populations. This underrepresentation of diverse ancestries, particularly from African, Hispanic, and Asian populations, poses risks in the development of effective therapies and diagnostic tools for non-European groups. The paper emphasizes that more diverse representation is crucial for understanding genetic predispositions and health outcomes across all populations. The paper underscores the importance of addressing both genetic and sociocultural determinants to ensure that research findings benefit all communities (3). Another study looking at national cancer cell line databases and 3D model cancer repositories found that a vast majority of these primary-human derived cellular resources were of European and/or unreported genetic ancestry (18). The authors of this work highlighted that there should be intentional design in preclinical cancer tissue engineering, especially as it pertains to the development of efficacious therapeutic outcomes. All of these analyses underscore the deficits in diversity that these fields operate under. However, they are limited to certain timeframes and particular parameters due to their arduous nature.

Traditional methods of manually extracting ancestry-related information from studies often involve tedious processes such as reading through papers, identifying relevant data, and categorizing it based on ancestry. These methods are labor-intensive and prone to human error and bias, especially when dealing with large datasets. Automated techniques like regular expressions (regex) have been introduced to expedite this process by searching for specific patterns or terms related to ancestry. However, regex-based methods are limited because they rely on predefined patterns and cannot interpret the broader context or meaning of the information (19). For instance, the format and naming conventions of cell lines or primary cell cultures often vary across studies, making it difficult for regex to capture relevant ancestry information accurately (20). Regex and similar automation techniques fail to comprehend the deeper meanings behind text, such as identifying the ancestry of a cell line or primary cell culture based on its source, especially when this information is embedded in complex or varied phrasing. This is where natural language processing (NLP) models like ChatGPT become invaluable. NLP models can not only detect keywords but also understand the context and meaning behind the information, enabling them to extract ancestry data more accurately and efficiently, regardless of the format or terminology used. This ability to comprehend meaning makes NLP models essential for scalable and inclusive research in this area.

Our work seeks to expedite the interrogation of ancestry distribution and representation in various fields through the establishment of an AI-assisted extraction tool compared to a manually extracted ancestry reporting database. This meta-analysis builds upon a previous study conducted with additional nuance in analyzing reporting practices across journals (17). We conducted a meta-analysis of 7 prominent bioengineering journals. From these journals, we pooled articles that mention use of some human-derived primary sample or cell line. We further analyzed this pool of articles to consolidate information related to reporting practices and the ancestral background of the cells. If the ancestral background of the samples were derived from the text of the article it was categorized as *Ancestry reported.* If the ancestry of the samples was able to be determined through a secondary source, they were considered as *Ancestry available*. If ambiguity in writing or cell sourcing left no means of determining ancestral background, the source was considered as *Ancestry not available*.

We then implemented a custom algorithm on this manually extracted database. This algorithm utilizes Generative Pre-trained Transformer-4 omni (GPT-4o) to automate data collection and processing. GPT-4o is a large language model pre-trained on an extensive corpus of internet text, enabling it to generate useful outputs based on any input text and/or image modalities. The implementation of this model in the study workflow is a five-step process. Initially, the text is extracted from research articles and broken into small chunks of text. These chunks are then fed to the model sequentially and the model is prompted to extract primary cell cultures and cell lines. After that, the model is prompted to extract any information about their ancestry. This tool then goes through another iteration of the refinement process to remove any unwanted or unrelated output that the model might add. Using this information and web crawling, the program makes two decisions: first if the ancestry of the given cell line or primary cell culture is available on a secondary source, and second if the ancestry of the cell culture is reported by the authors of the articles. It also extracts the correct genome ancestry from the internet using web crawling. Subsequently, this output can be analyzed to discern ancestral reporting practices. This tool has been named TRACE and is hosted in a collaborative web-platform offered in Additional Information.

Our research ultimately revealed that ancestry is consistently underreported, aligning with previous work within and in adjacent fields. When ancestry information was available, the majority of cell lines were of White/European descent. Importantly, within this database, ancestry availability of cell lines was demonstrated to be highly prevalent. Leveraging this manually extracted database we were able develop and validate an automated tool to streamline the workflow, enabling feasible and reproducible analyses. Manual filtering and data extraction, which are time-consuming and resource-intensive, were identified as bottlenecks in the project. By reducing the time and cost associated with these steps, our workflow becomes more efficient and scalable for future inquiries and inquiries within adjacent fields.

## 2 Methods

Upkeep and evaluation of ancestry reporting as the field progresses is imperative to address current and future gaps in transformative science. The assistive screening tool introduced in this work allows for consistency in this evaluation and alleviates aspects of manual ancestral extraction. Utilizing the GPT-4o model for text extraction represents a significant advancement over traditional natural language processing methods, as it simplifies the process through ChatCompletion, as well as offering a higher degree of accuracy in the generated responses. GPT-4o, developed by OpenAI, is a highly powerful language model built upon a deep neural network architecture called a transformer model. This architecture leverages multiple stacked self-attention layers to process and generate human-like text. GPT-4o benefits from extensive training on vast amounts of diverse text data, enabling it to learn intricate patterns, comprehend contextual nuances, and produce coherent and contextually relevant outputs. The model follows a semi-supervised learning approach, initially training a large unsupervised corpus of text and subsequently fine-tuning itself through self-supervised learning using human and model feedback.

The GPT-based tool, TRACE, offers several significant advantages over traditional and regex-based methods for ancestry reporting in meta-analysis. Unlike regex, which relies on fixed patterns, this tool understands context, enabling it to accurately interpret and extract ancestry data even when phrasing or terminology varies across studies. It is highly scalable, capable of processing large datasets rapidly, which saves significant time compared to manual extraction. By utilizing natural language processing, the tool achieves higher accuracy in identifying human cell lines and primary samples, even when information is presented in complex or indirect language. Additionally, it automates the extraction of ancestry data, reducing human error and bias, while its dynamic web crawling feature allows it to retrieve missing ancestry information from secondary sources like cell line databases. The tool also enhances reproducibility, ensuring that the data extraction process can be consistently repeated across different studies, improving the reliability of meta-analyses. Its inclusive approach to data extraction ensures that studies from diverse backgrounds are better represented. Finally, by automating labor-intensive processes, the tool frees researchers to focus on higher-level analysis, making it a highly efficient and robust solution for ancestry reporting in bioengineering research. While this process significantly improves efficiency and accuracy, especially compared to manual extraction methods, the tool occasionally falls short. In about 6%–7% of the papers, TRACE fails to extract all relevant cell culture and ancestry information. This issue arises when the text contains complex or unconventional phrasing that GPT-4o struggles to interpret. For instance, when studies present ancestry data in non-standard formats or use rare terminology, TRACE's ability to capture the full scope of information becomes limited.

Future applications of the assistive screening tool can be expanded to evaluate other fields of studies related to health inequities while providing an opportunity to answer other ancestry-related inquiries in medicine. The tool, which can be accessed on GitHub (https://github.com/lab-smile/TRACE), offers a flexible framework that can be adapted for these extended applications. For example, the use of this study can be modified to look at what ancestral backgrounds of biological constituents most represented by other particular pathologies or health disparities. This would also allow the field to interrogate if biological samples used to study a particular health issue is representative of the demographic impacted by that issue. While the scope of this study does not by itself interrogate the impacts that ancestry has on biomedical research, it postulates on precedents of other fields that have demonstrated an overrepresentation of White/European demographics (4, 5). Ancestry can no longer be undervalued and capturing the impacts it has on the diverse response amongst cells and tissues may play a pivotal role in building upon personalized medicine.

### 2.1 Journal selection and rationale for timeframe

The 6-month timeframe for this study took place from 01 July 2021 to 31 December 2021. This allows for comparisons to be drawn with similar prior meta-analyses (14) that utilized the same monthly timeframe. Additionally, this study also mirrored the same journal selection as the previous meta-analysis, apart from three journals. Journals were selected based on their high-impact factor (Supplementary Figure 1) and their focus on leveraging biomaterials for *in vitro* cultures/modeling. High-impact journals were utilized due to the greater likelihood of mentioning the source of cells or samples used. The journals that were used in this study are Nature Biotechnology, Nature Biomedical Engineering, Science Translational Medicine, Advanced Healthcare Materials, Journal of Translational Medicine, Lab on a Chip, and Journal of Biomedical Materials Research. The three journals that were excluded (ACS Biomaterials Science, Frontiers in Bioengineering and Biotechnology, Scientific Reports) had the largest number of articles published (>1,000 article hits for the timeframe of interest) as well as lower impact factors. The low impact score and article quantity combined with the expanded nuance, analysis, and development of an assistive screening tool that this study takes on is the reasoning behind the journal exclusion. Larger volume journals were excluded to ensure the manual validation process remained feasible. Even with this exclusion, the database developed within this work exceeds previous work, with the evaluation of 326 articles (compared to 202 from a prior study) and 743 incidences of human sample use (compared to 341 from a prior study) (14). This expansion makes this work the largest meta-analysis of its kind to date.

### 2.2 Inclusion and exclusion criteria

**Figure 1:**
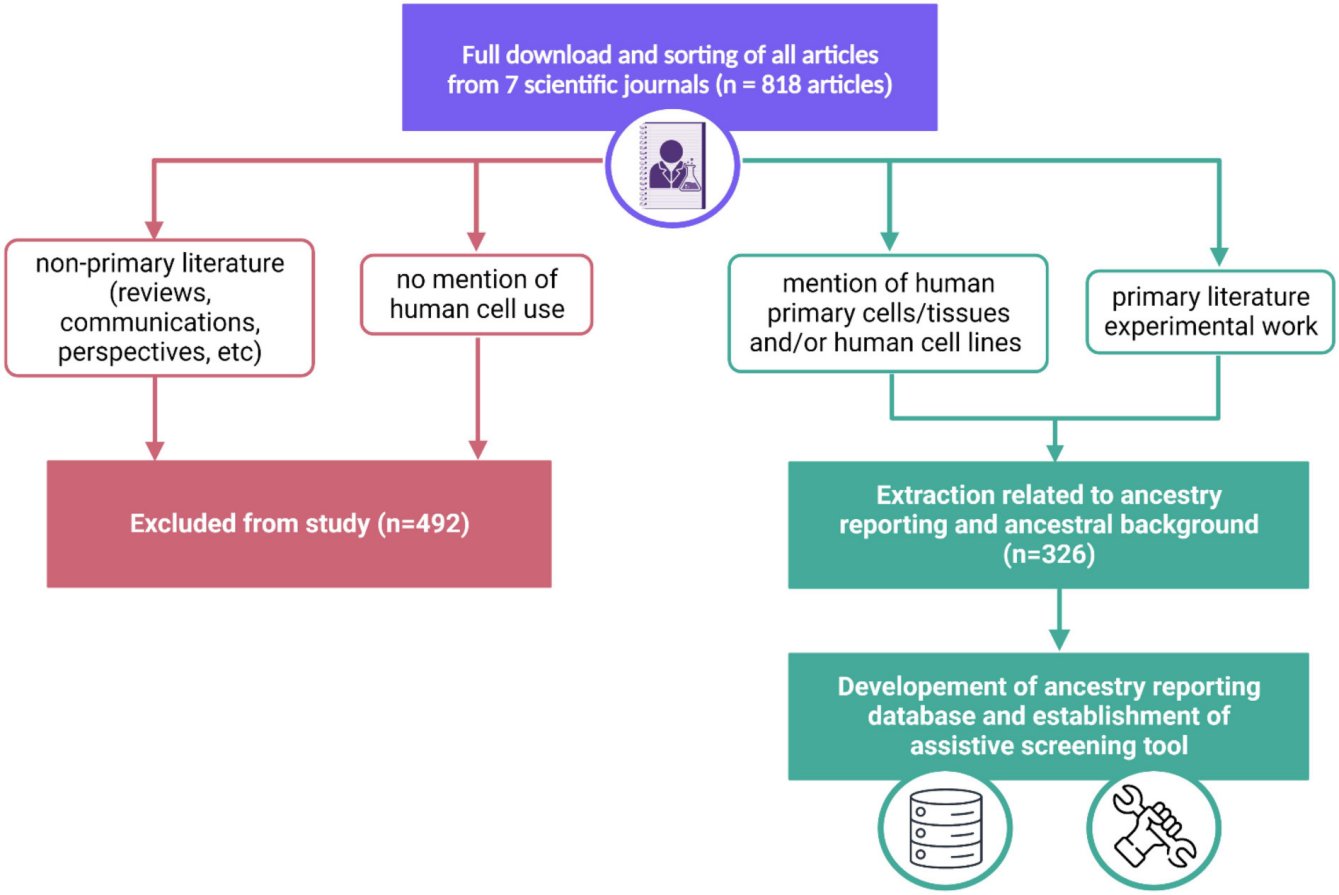
Workflow of manual analysis of 818 articles within 7 journals. Exclusion (pink) and inclusion (green) pathways are delineated to demonstrate the ultimate establishment of an ancestry reporting database and assistive screening tool.

All articles were downloaded from the journals during the above timeline (*n* = 818). These articles were then sorted based on the exclusion and inclusion criteria listed below and presented in Figure 1.

Inclusion criteria:

• Primary scientific literature
• Mention some use of human cell line, primary cell culture, and/or other primary human biological constituent (i.e., whole blood or tissue)

Exclusion criteria:

• Non-primary scientific literature (i.e., reviews, communications, perspectives, etc.)
• Only mentions the use of animal cells or tissues

Based on the criteria, 492 articles were excluded and had no further downstream analysis conducted. All articles that met the inclusion criteria were inputted into the notion workspace for further downstream analysis (21). These articles were identified within the database through their name and digital object identifier. For articles that mention the use of both animal and human samples, only the human ones were added to the notion database. Every mention of a given cell, cell line or tissue warranted its own incidence within the database. For example, HeLa cells were utilized 28 distinct times warranting 28 incidences within the database.

### 2.3 Ancestry reporting practices categories

All included articles were sorted into different reporting practices, defined in Figure 1. These categorized practices included *Ancestry not available, Ancestry available* and *Ancestry reported*. Articles can possess more than one reporting practice if there is some ancestry availability for some of the samples used, but not for others. *Ancestry not available* refers to articles that had mention of human cell or tissue use, but the ancestral background of those constituents was indiscernible. *Ancestry available* includes articles in which human samples' ancestral background could be traced through a secondary source. *Ancestry reported* refers to articles that directly report on the ancestral background of the human cell or tissue used.

### 2.4 Ancestry categories

Ancestry for cell lines and primary samples mentioned in the text was determined by first making a pass through the article to see if it was provided within the text. If not provided directly in the text, then a secondary source was utilized to extract the ancestry of the cell or tissue in question. Oftentimes this secondary source was the cell line database cellosaurus.org. Occasionally vendor websites (e.g., atcc.org) or other cell line databases (e.g., hpscreg.eu) were used. To expand on previous work all cell lines and primary samples were sorted into different ancestral categories based on three different ancestral groupings:

1. Ancestral groupings based on the United States census were used and included Black or African American, White, Asian, Native Hawaiian or Pacific Islander, American Indian, and Two or more races.
2. The second grouping that was used is based on genotyping studies (22–24) and includes the following: African, European, Central/South Asian, East Asian, and Mixed Ancestry. Several cell lines had specific ancestral breakdown. For consistency, we determined that whatever ancestral background was >60% would be the assigned ancestry for that cell line. However, if it was <60% then the cell would be considered to be of “Two or more races” and/or “Mixed Ancestry.”
3. The final grouping implemented is noting the ancestral labeling used when extracting either from a given primary or secondary source. For example, a study may report that primary cells were taken from a “Han Chinese” patient. Therefore, that descriptor would be reported within a table. All these groupings for each sample are noted in the supplement (Supplementary Tables 1, 2).

Ancestry for this database was categorized in three separate ways to minimize limitations associated with different practices of assigning ancestral background and to appreciate the nuance more accurately in reporting ancestry, genotyping, and allowing for comparison with previously reported studies.

### 2.5 Development of TRACE

To develop an AI assistive tool for extraction, we leverage GPT-4o for integration with text and meta analysis. Our code starts by extracting the text from each PDF file and dividing it into smaller chunks for subsequent “reading”. The size of the chunk is dependent on the token count, similar to character counts, however, in this case, tokens are a unit of semantic meaning processed by the language models. Our tool ensures that each chunk contains fewer than 4,000 tokens.

The text chunks are submitted to the OpenAI's API using a ChatCompletionRequest with a prompt that requests information about cell types, formatted as an array. To ensure consistent and deterministic responses, the model's temperature is set to zero. The cell culture outputs are stored in a way that corresponds to the specific text chunks from which they were extracted. These initial results from the API are then refined by passing all outputs through the GPT-4o model to filter out entries that may not represent a cell line or primary cell culture. The refined outputs, along with their corresponding text chunks stored in a dictionary, are fed back into the model to extract any available information about the ancestry of the cell cultures from these text chunks.

Additionally, the tool crawls the web and queries the Cellosaurus website to find the closest match for each culture. If a match is found with a similarity score greater than 50%, the tool further searches for any available ancestry information. If no match exceeds the 50% threshold, the tool does not provide ancestry details.

The results obtained from the API are added to a table that includes the following columns: Article ID, cell culture found, cell name identified on the web, ancestry available, reported decision, ancestry information identified by GPT, and ancestry information found on the web. This side-by-side comparison allows for easy validation of the output's accuracy and consistency. The table can then be used to populate the database manually to ensure the validity of the model's output, while still cutting down on the reading and analysis time required for individual processing by transforming the extraction task into a validation task. The table can also be used for downstream tasks which can improve the project in further iterations.

### 2.6 Qualifiers and limitations to study

This study is subject to the several limitations listed and explained below:

• Race and ethnicity are limited terms that are dependent on their sociocultural setting. For that reason, we went with a broad usage of the term ancestry. We describe ancestry within this work as any background lineage claim of an individual. This range includes, but is not limited to, the following: geographical, genetic, cultural, self-reported, and perceived ancestral claims. This study does not seek to redefine ancestry.
• Mislabeling of the ancestral background of cell lines and cell line contamination. Studies, particularly related to cancer, have found mislabeling of ancestral backgrounds for several cell lines (25). Additionally, it is well-known that mammalian cell lines are subject to cross-contamination amongst one another, and investigators rarely test for this when conducting experiments. A major contributor to this cross-contamination is the HeLa cell line (26).
• Human error in manual extraction.
• Timeframe and journal selection. This work looks at a particular timeframe and sample of journals and therefore is limited by this sampling.
• Admixed vs. Mixed Ancestry category. The Mixed Ancestry category is not typically seen in genotyping studies, however, for this study, it took the place of admixture. Claims to admixture, which is the formulation of new genetic populations, could not accurately be made with human sampling of individual donors.

## 3 Results

### 3.1 Ancestry was found to be mostly underreported among bioengineering journals

**Figure 2:**
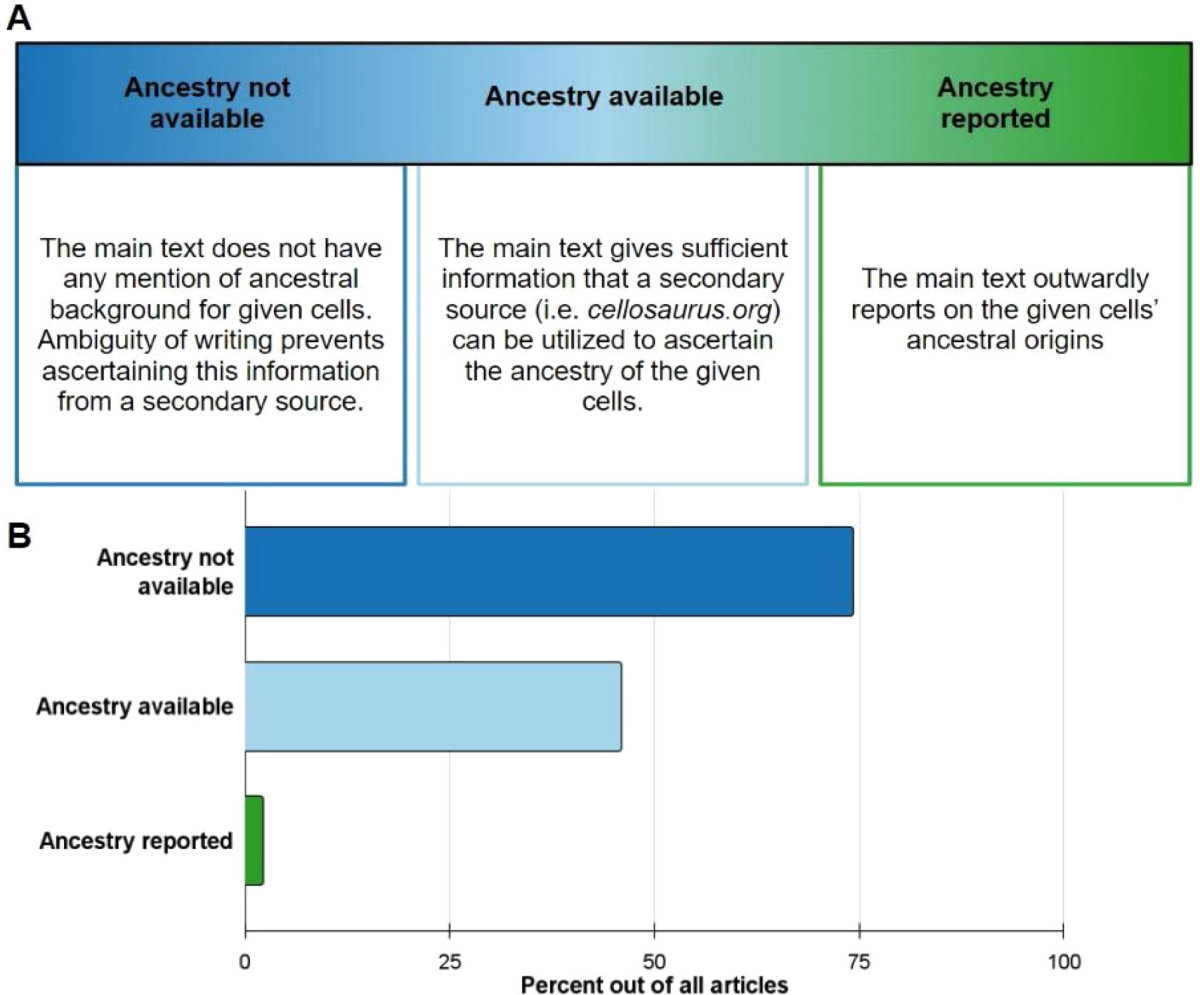
Ancestral reporting practices amongst 326 articles within 7 journals. **(A)** Description of the following ancestry reporting categories: ancestry not available, ancestry available, and ancestry reported. **(B)** Breakdown of all articles into respective reporting practices. Ancestry not available (dark blue) made up 74.3%, Ancestry available (light blue) 45.9%, and ancestry reported (green) 2.1%. Articles can fall under multiple reporting practices for the different cells/tissues they used.

Among the seven journals analyzed, 326 out of 818 articles consisted of instances of human cell or tissue use. These articles were cataloged and sorted into different reporting practices including *Ancestry not available*, *Ancestry available*, and *Ancestry reported* (Figure 2A). Of these practices, 74.3% of articles fell under *Ancestry not available* (Figure 2B). *Ancestry available* is the second category that most articles fell under at 45.9% (Figure 2B). Direct ancestry reporting within the main text was found to be extremely limited, occurring in only 2.1% of all analyzed articles (*n* = 9). All these direct reporting incidents occurred with primary samples as summarized in Figure 2B.

### 3.2 Most cells and tissues that had ancestry available were of White/European descent

**Figure 3:**
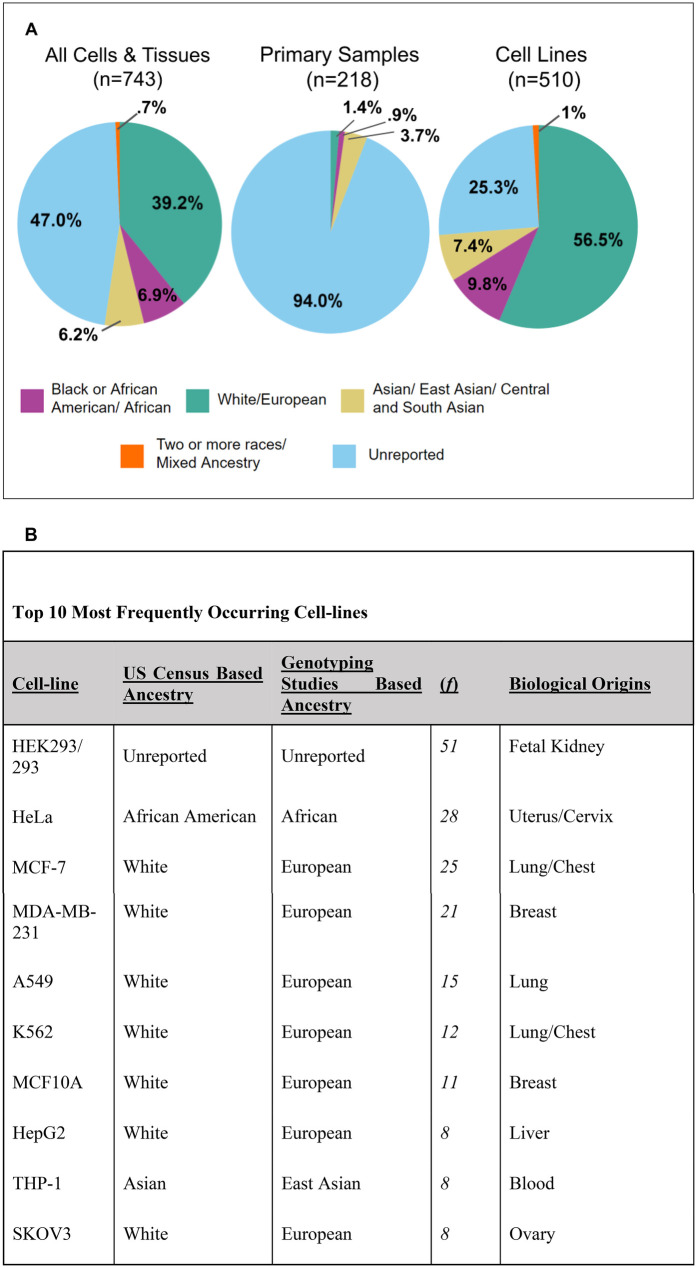
Breakdown of 743 incidences of human cell and tissue use into ancestral groupings. **(A)** Pie charts representing the entire human cells and tissues database (*n* = 743), primary samples (*n* = 218), and cell lines (*n* = 510). Some samples were unspecified as being a primary cell or cell line and were not displayed (*n* = 15). Ancestral categories include the following: Black or African American/African, White/European, Asian/East Asian/Central and South Asian, and Two or more races/Mixed Ancestry. **(B)** Table of top 10 most frequently occurring cell lines listed from highest to lowest. The table includes information related to the cell line's ancestry, frequency in the database, and biological origins.

Many of these articles used multiple types of cells/tissues and every instance of human cell/tissue usage was recorded. In total, there were 743 incidences, 510 of which were immortalized cell lines, 218 were human primary samples, and 15 were unspecified. A large portion of these samples had no ascertainable ancestry (47% of all samples). Of all of the samples, 39.2% were of White/European descent (Figure 3A). Of the samples that had ascertainable ancestry, ∼74% were of White/European descent. Black or African American/African (6.9%), Asian/East Asian/Central and South Asian (6.2%), and Two or more races/Mixed Ancestry (0.7%) were significantly underrepresented in the total sample usage (Figure 3A). American Indian and Native Hawaiian/Pacific Islander did not occur within the database. However, it is important to note, that some American Indian ancestral breakdown was present for some cell lines; the genotype percentage of American Indian never took on a majority percent of the cell line in question.

Cell lines made up the majority (∼67%) of this database (Supplementary Figure 1A). We identified 212 distinct human cell lines across 326 included articles. Cell lines demonstrated a similar pattern as the overall database for ancestry representation, but only 25% of all cell line incidences were unreported (Figure 3A). White/European represented the majority of cell lines (56.5%) used (Figure 3A). All other ancestral backgrounds were severely underrepresented. Black or African American/African accounted for 9.8% of the cell lines used, Asian/East Asian/Central and South Asian accounted for 7.4%, and Two or more races/Mixed Ancestry accounted for 1% (Figure 3A). Additionally, of all Asian-represented cell lines (including Asian, East Asian, and Central and South Asian), only one (the FaDu cell line) was of Central and South Asian descent. Figure 3B consists of the top ten most frequently occurring cell lines. Despite HeLa, a cell line of Black or African American/African ancestry, being the second most commonly used, most of the Top 10 frequently used cell lines were of White/European descent (7 out of 10) (Figure 3B). An overwhelming majority of primary samples had ancestry unreported (94%) (Figure 3A). These numbers closely reflect previous studies which similarly found that 94.2% of primary sample usage and 25.3% of cell line usage had unreported ancestry (14). While the study represents a 6-month period for seven bioengineering journals, we believe the results likely reflect the broader landscape of reporting practices in the field and are consistent with prior studies (5, 14).

### 3.3 Establishment of TRACE

**Figure 4:**
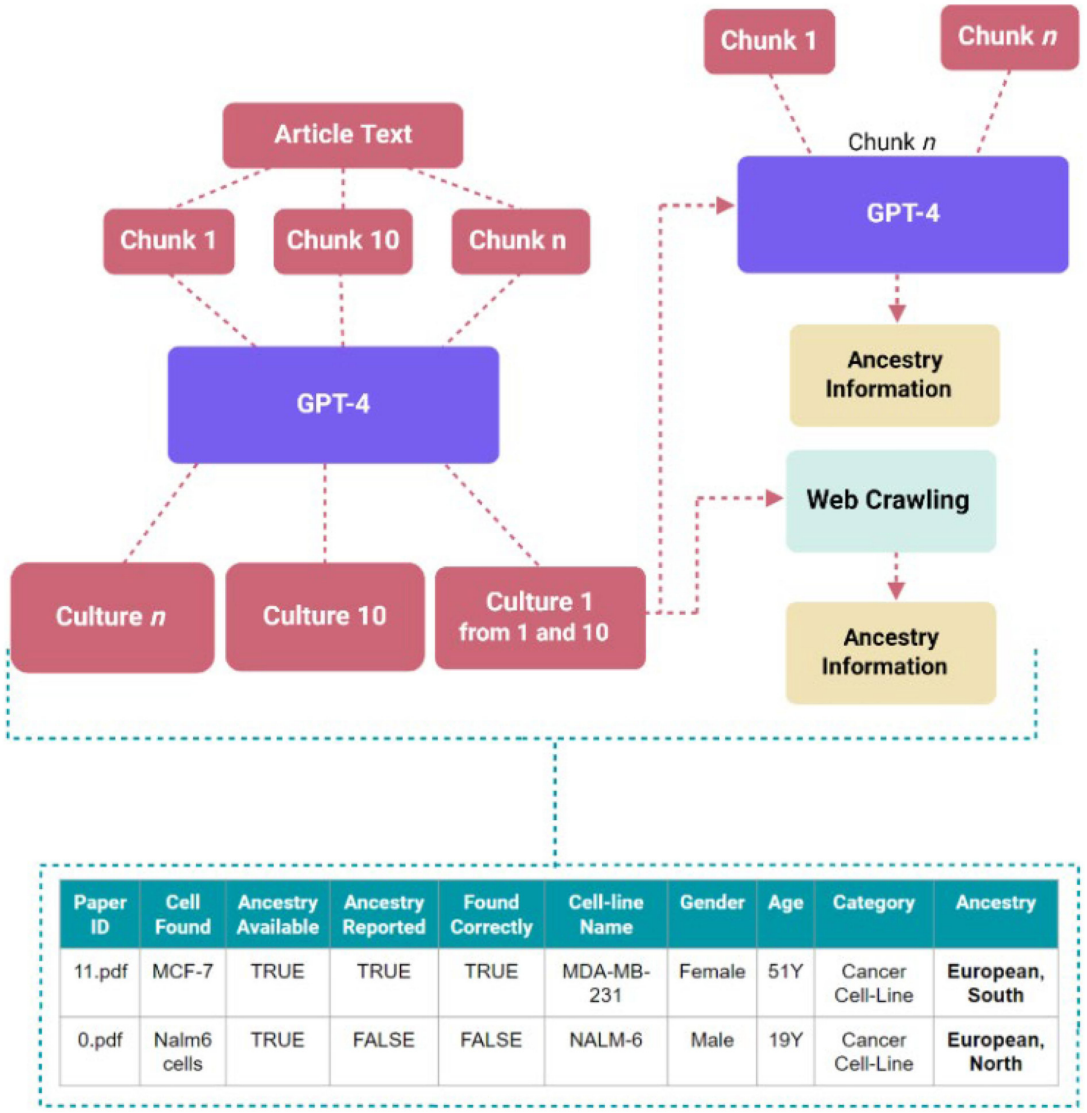
Workflow and architecture for assistive screening tool, TRACE, and example of the table for facile ancestry reporting extraction. General workflow of automated extraction.

The workflow, as shown in Figure 4, begins by providing a collection of research articles for the GPT-4o model in a folder. The model then extracts the text from these articles, which serves as the input for further analysis. The settings for the prompt contain values for the user, system, and assistant roles, including the prompts given to the application programming interface (API) call. This approach allows the model to identify instances where these entities are mentioned in the text. Initially, the prompts focus on identifying human samples, which helps to exclude non-human cells and cell lines. Subsequently, these results are refined using a different prompt that filters out anything that is not specifically human cell lines or cell cultures. This two-step process helps the tool to be both sensitive and specific. Once the instances of human samples are identified, the model goes a step further and determines whether each article provides information on the ancestry of these entities. For primary cell cultures like T cells, which do not have any definitive ancestry on Cellosaurus website, will be categorized as “not reported” if an article fails to mention its ancestry. For cell lines, the tool will check whether the ancestry information is reported in the article and on the Cellosaurus website to determine the category. The output contains a list of the articles, with relevant text, and the associated information of cell types and ancestry mentioned. For cell lines and primary cell cultures not mentioned in the article, the tool utilizes web crawling to explore online cell line databases and extract information about the ancestry of the cells, even if this data is not directly reported in the article.

The assistive screening pipeline helps reduce the time required for the preliminary processing of the data by turning the filtering and extraction tasks into predominantly validation tasks. Rather than manually reading the content of each paper to identify mentions of cell type or ancestry, often with thick blocks of texts obscuring casual and sparse mentions, mentions in the lists generated by the pipeline in CSV format are assessed. This can then be validated by comparing the extracted values against the original chunk of text next to it. This is a streamlined process, allowing for more efficiently generated data for downstream processing.

### 3.4 Evaluating TRACE against manually extracted dataset

TRACE was rigorously tested against a human-curated dataset to assess its ability to identify cell cultures and accurately determine their ancestry. While TRACE accurately identified many exact matches, its raw outputs also included matches that were semantically correct but not literal string matches. To account for these, we introduced the category of “Good Matches Found”, defined as predicted cell culture names that achieve a string similarity score of ≥50% with the reference names using a normalized Levenshtein distance. This threshold was empirically selected to balance sensitivity and specificity, allowing TRACE to capture meaningful variations in naming while filtering out unrelated results. Incorporating this category allows for a more realistic assessment of TRACE's practical utility in handling heterogeneous and inconsistently reported data across the biomedical literature. While TRACE effectively extracted relevant information, its raw outputs contained loosely related results and occasional misclassifications due to inconsistencies in how ancestry is reported across studies. To improve accuracy, we implemented a post-processing step to refine the tool's ability to detect and classify ancestry. This process involved filtering out ambiguous matches, removing misclassified entries, and improving the precision of extracted ancestry data. Before refinement, TRACE achieved an Ancestry Accuracy of 81.82%, with 163 exact matches out of 431. After refinement, accuracy slightly adjusted to 80.91%, with 165 exact matches, but the proportion of “Good Matches Found” improved from 79.12% to 80.01%, demonstrating that while overall accuracy remained stable, TRACE became more precise in identifying relevant results as well as instances of model hallucination. To enhance precision, we implemented a post-processing step that filtered out predicted cell cultures not explicitly mentioned in the source text. This refinement reduced false positives and improved the semantic relevance of matches by ensuring that only grounded, text-supported predictions were retained. Prior to post-processing, TRACE achieved 163 exact matches with the manually curated dataset. Although the overall accuracy remained largely consistent after refinement with slight improvement, reflecting improved alignment between predictions and source content.

Beyond accuracy, TRACE's automated process often retrieved an excessive number of extra outputs due to the diverse ways cell cultures are referenced in literature. Many cell lines appear under multiple names, leading to redundant matches, while some extracted results were only loosely related to the expected cell cultures. To address this, we developed a curation step that grouped duplicates and clustered highly similar cell culture references, significantly reducing noise while retaining meaningful extractions. Before curation, TRACE produced an overwhelming 4,123 extra outputs for 431 expected matches, making manual review difficult. After curation, the number of extra outputs was reduced to 1,963, demonstrating that grouping similar entries and filtering redundant results improved the efficiency of the tool without compromising sensitivity. While the high number of extra outputs may seem like a drawback, it is actually an advantage over manual extraction, as TRACE captures every single cell culture reference present in the text. This comprehensive extraction ensures that no relevant data is overlooked, unlike human extraction, which may miss less explicitly mentioned cultures. Combined with the curation process, this enhances TRACE's effectiveness in cell culture identification, making it a highly efficient and thorough tool for ancestry reporting in biomedical research.

The combined effect of post-processing and curation significantly enhanced TRACE's performance. An Overall Similarity Score was calculated as the average string similarity (*via* normalized Levenshtein distance) between each predicted cell culture and its closest corresponding match in the manually curated dataset. This score improved from 77.98 to 81.98, highlighting better alignment with the manually extracted dataset. The final accuracy metrics showed an Ancestry Accuracy of 80.91%, 165 exact matches, and 80.01% good matches found after refinement, while the number of extra results was reduced by more than half. These results demonstrate that TRACE, when coupled with systematic post-processing and curation, is a powerful tool for ancestry reporting, capable of balancing sensitivity and specificity to provide accurate, relevant, and actionable outputs for large-scale biomedical research.

## 4 Discussion

Upkeep and evaluation of ancestry reporting as the field progresses is imperative to address current and future gaps in transformative science. The assistive screening tool introduced in this work allows for consistency in this evaluation and alleviates aspects of manual ancestral extraction. Utilizing the GPT-4o model for text extraction represents a significant advancement over traditional natural language processing methods, as it simplifies the process through ChatCompletion, as well as offering a higher degree of accuracy in the generated responses. GPT-4o, developed by OpenAI, is a highly powerful language model built upon a deep neural network architecture called a transformer model. This architecture leverages multiple stacked self-attention layers to process and generate human-like text. GPT-4o benefits from extensive training on vast amounts of diverse text data, enabling it to learn intricate patterns, comprehend contextual nuances, and produce coherent and contextually relevant outputs. The model follows a semi-supervised learning approach, initially trained on a large unsupervised corpus of text and subsequently fine-tuning itself through self-supervised learning using human and model feedback.

The GPT-based tool, TRACE, offers several significant advantages over traditional and regex-based methods for ancestry reporting in meta-analysis. Unlike regex, which relies on fixed patterns, this tool understands context, enabling it to accurately interpret and extract ancestry data even when phrasing or terminology varies across studies. It is highly scalable, capable of processing large datasets rapidly, which saves significant time compared to manual extraction. By utilizing natural language processing, the tool achieves higher accuracy in identifying human cell lines and primary samples, even when information is presented in complex or indirect language. Additionally, it automates the extraction of ancestry data, reducing human error and bias, while its dynamic web crawling feature allows it to retrieve missing ancestry information from secondary sources like cell line databases. The tool also enhances reproducibility, ensuring that the data extraction process can be consistently repeated across different studies, improving the reliability of meta-analyses. Its inclusive approach to data extraction ensures that studies from diverse backgrounds are better represented. Finally, by automating labor-intensive processes, the tool frees researchers to focus on higher-level analysis, making it a highly efficient and robust solution for ancestry reporting in biomedical research.

While the tool offers many advantages, both its limitations and those of the study itself should be acknowledged. In about 6%–7% of the papers, TRACE fails to extract all relevant cell culture and ancestry information. This issue arises when the text contains complex or unconventional phrasing that GPT-4o struggles to interpret. For instance, when studies present ancestry data in non-standard formats or use rare terminology, TRACE's ability to capture the full scope of information becomes limited. Additionally, the study focuses on high-impact journals and those with smaller, more manageable volumes. High-impact journals were selected for their presumed greater robustness, which increases the likelihood of reporting on cell/sample origin. Larger volume journals, such as Scientific Reports, were excluded for feasibility purposes in the manual extraction done within the study. Another limitation of the study is the use of ancestral nomenclature, which is inconsistent and can lead to misinformation in scientific literature. Issues related to ancestral designations are discussed in greater detail in the Methods section under “Qualifiers and Limitations of the Study.”

Another limitation of TRACE is the accessibility of GPT-4o, as its full functionality requires a paid subscription. However, users can readily access trial versions of GPT-4o mini and GPT-4o to evaluate TRACE's suitability for specific studies. Currently, TRACE relies on the OpenAI API to utilize both GPT-4o and GPT-4o-mini for language understanding tasks. While we recognize the constraints of API-based access, we are actively exploring alternatives that support open-source and local solutions, such as LLaMA and Mistral, which can perform inference offline. In the future we plan to release a companion script that will integrate freely available models through Hugging Face pipelines or allow for local deployment. This will help make TRACE more accessible to a broader community. Additionally, TRACE's modular architecture is designed to easily accommodative the backends of alternative large language models. Although our initial focus has been on cell line ancestry, the pipeline is highly adaptable to other entity extraction tasks such as demographic reporting and sample metadata. Future studies can continue to explore its applicability across a variety of use cases.

Future applications of the assistive screening tool can be expanded to evaluate other fields of studies related to health inequities while providing an opportunity to answer other ancestry-related inquiries in medicine. The tool, which can be accessed on GitHub (https://github.com/lab-smile/TRACE), offers a flexible framework that can be adapted for these extended applications. For example, the use of this study can be modified to look at what ancestral backgrounds of biological constituents most represented by other particular pathologies or health disparities. This would also allow the field to interrogate if biological samples used to study a particular health issue is representative of the demographic impacted by that issue. While the scope of this study does not by itself interrogate the impacts that ancestry has on biomedical research, it postulates on precedents of other fields that have demonstrated an overrepresentation of White/European demographics (4, 5). Ancestry can no longer be undervalued and capturing the impacts it has on the diverse response amongst cells and tissues may play a pivotal role in building upon personalized medicine.

## Data Availability

The datasets presented in this study can be found in online repositories. The names of the repository/repositories and accession number(s) can be found in the article/Supplementary Material. The code for this project is hosted at https://github.com/lab-smile/TRACE.
