# Supplementary material for: TRACE: applying AI language models to extract ancestry information from curated biomedical literature
Source: Front Digit Health. 2025 Sep 19;7:1608370. doi: 10.3389/fdgth.2025.1608370 (PMC12491185; doi:10.3389/fdgth.2025.1608370)
Supplement: Supplementary file 1 [file Datasheet1.pdf]

A

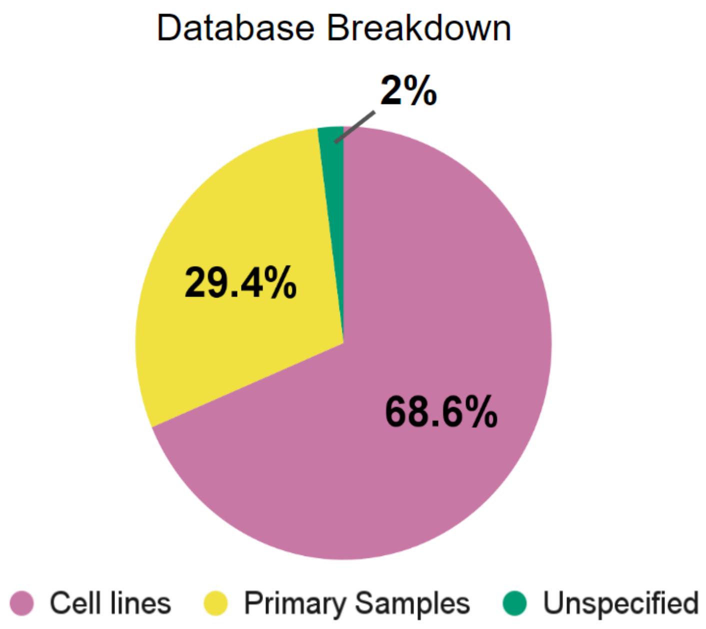

B

Journal	Impact Factor (IF)	Countries of Origin of Editorial Board
Nature Biotechnology	**68.2**	Britain, Germany, China
Nature Biomedical Engineering	**29.2**	United States, Britain, Germany
Science Translational Medicine	**19.3**	United States, Britain, Germany
Advanced Healthcare Materials	**11.1**	United States, Germany, Britain, Hungary, China, India, Australia, Turkey
Journal of Translational Medicine	**8.4**	United States, Germany, China, Spain, India, Qatar
Lab on a Chip	**7.5**	United States, Canada, France, South Korea, Japan, China, Netherlands
Journal of Biomedical Materials Research	**4.9**	United States, Australia, Singapore, China, Italy, Canada, Britain, Japan, South Korea, Russia, Germany

Supplemental Figure 1. Additional information regarding the database and journals included. **A)** Overview of all incidences of human sample use (n=743) divided by cell line (pink), primary samples (yellow) or if it was unspecified (green). **B)** Table demonstrating impact factor (from highest to lowest) as well as countries of origin for the given editorial boards.

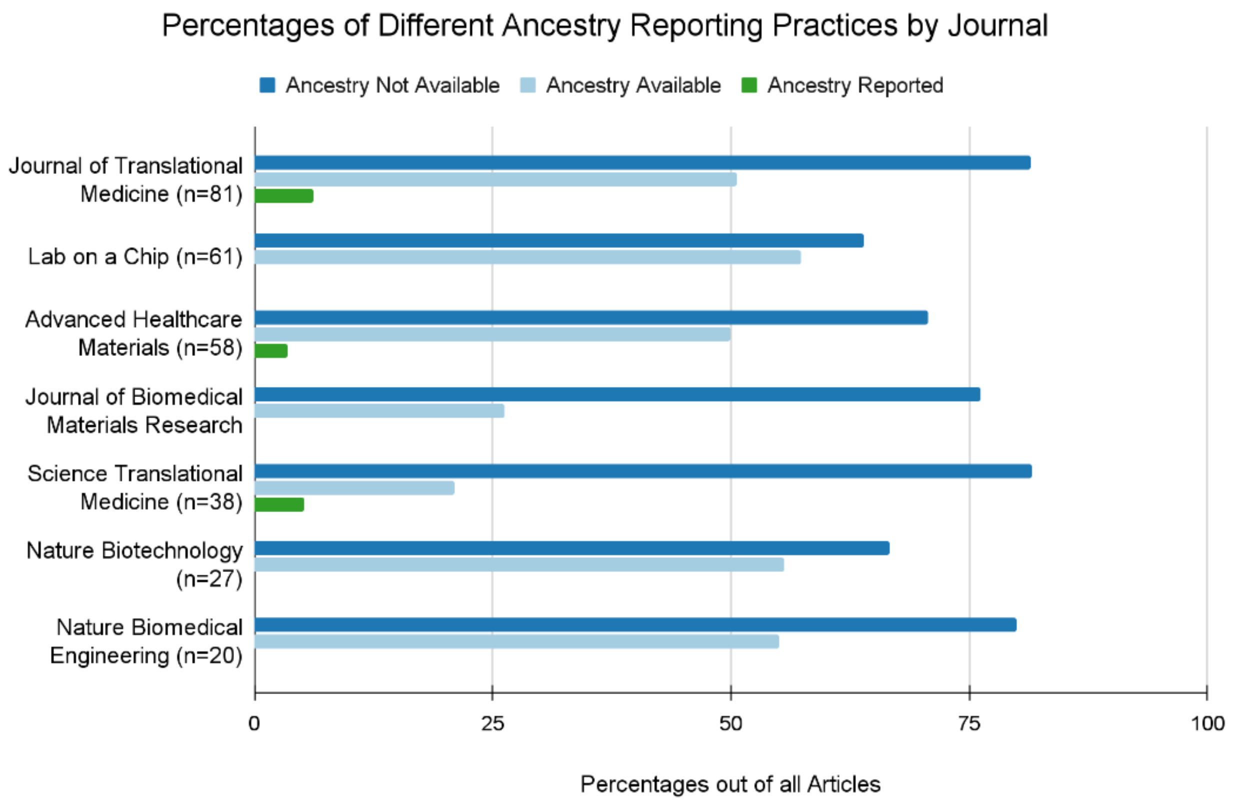

Supplemental Figure 2. Breakdown of all articles into respective reporting practices divided by journal. The following categories were used: *Ancestry not available* (dark blue), *Ancestry available* (light blue), and *Ancestry reported* (green). Articles can fall under multiple reporting practices for the different cells/tissues they used.

.

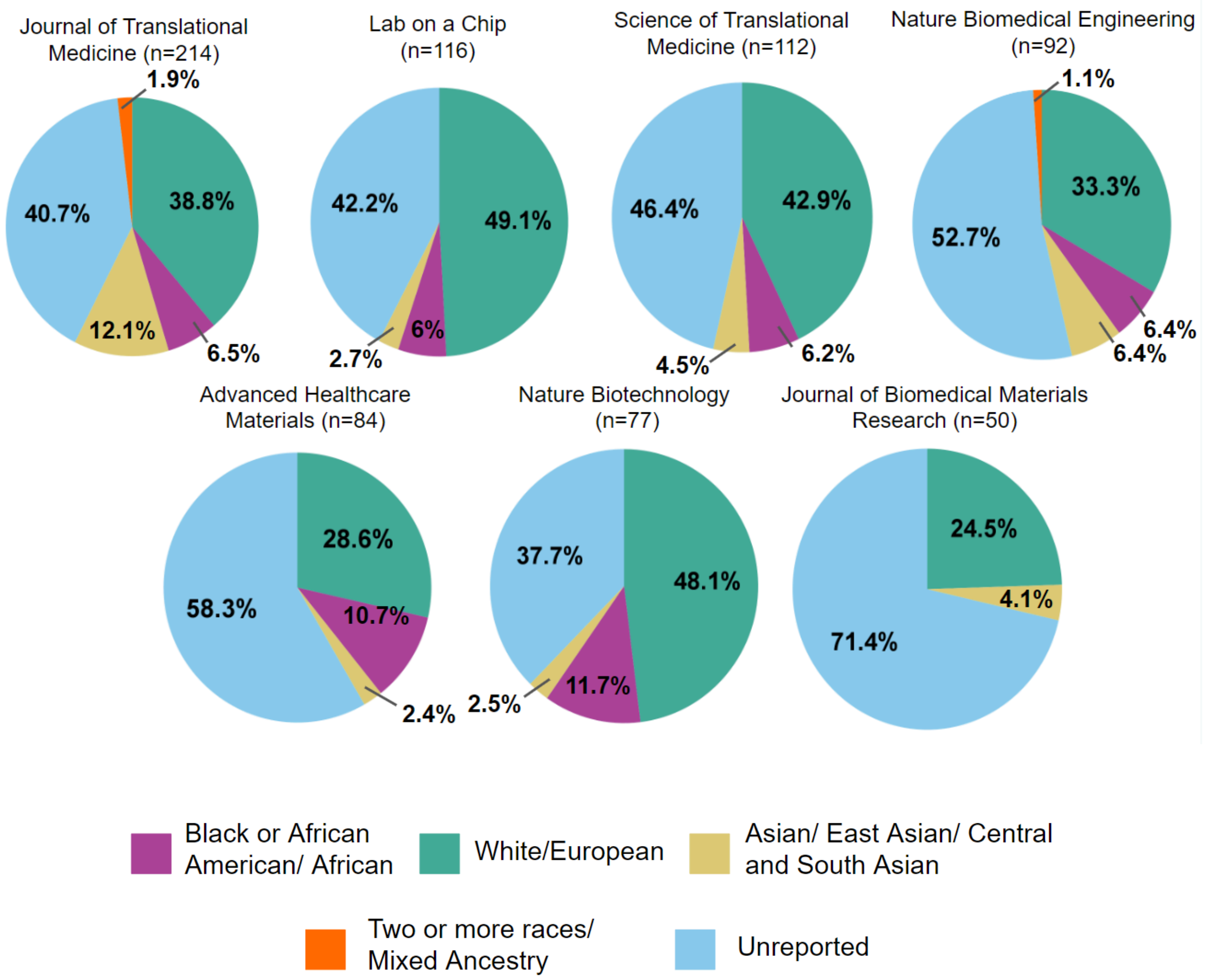

Supplemental Figure 3. Ancestral breakdown of human samples divided by journal. Ancestral categories include the following: Black or African American/African, White/European, Asian/East Asian/Central and South Asian, and Two or more races/Mixed Ancestry. If the ancestral category was not available for a human sample, then it was considered to be Unreported.

Ancestries of all Cell lines				
**Cell line**	**US Census Based Ancestry**	**Genotyping Studies Based Ancestry**	**Directly Stated in Primary or Secondary Source**	
1383D2 hiPSC	Asian	-	*Asian*	1
143B	Two or more races	Mixed Ancestries	*East Asian (4.02%), South Asian (37%), Southern European (58.98%)*	2
201B7 hiPSC	White	European	*Caucasian*	1
414C2 hiPSC	White	European	*Caucasian*	2
5637	Two or more race	Mixed Ancestries	*African (30.56%), Native American (.45%), Northern East Asian (40.31%), South Asian (.35%), Northern European (4.97%), Southern European (11.89%)*	1
786-O	White	European	*African (.77%), Northern East Asian (4.36%), North European (65.75%), Southern European (29.12%)*	2
A172	White	European	*Northern East Asian (2.05%), South Asian (1.21%), Northern European (68.98%), Southern European (27.77%)*	1
A2780	Black or African American	African	*African (93.08%), Northern East Asian (2.76%), Southern European (4.17%)*	1
A375	White	European	*African (.84%), Native American (.49%), Northern East Asia (1.69%), Northern European (67.22%), Southern European (29.75%)*	1
A431	White	European	*African (3.82%), Northern East Asian (7.84%), South Asian (6.03%), Northern European (17.15%), Southern European (65.16%)*	1
A498	White	European	*African (.37%), Northern East Asian (2.1%), Northern European (63.47%), Southern European (34.06%)*	1
A549	White	European	*Northern East Asian (.72%), Northern European (66.12%), Southern European (33.16%)*	15
ACHN	White	European	*Northern East Asian (.83%), Southern East Asian (1.52%), South Asian (1.78%), Northern European (70.53%), Southern European (25.34%)*	1

AGS	Two or more races	Mixed Ancestries	*African (3.44%), Native American (32.25%), Northern East Asian (5.38%), South Asian (.23%), Northern European (27.58%), Southern European (31.12%)*	1
AsPc-1	White	European	*Native American (.97%), Northern East Asian (3.23%), Northern European (58.23%), Southern European (37.57%)*	2
BALL-1	Asian	East Asian	*Northern East Asian (81.09%), Southern East Asian (18.9%)*	1
BCG-823	Black or African American	African	*African (65.99%), Native American (.77%), Northern East Asian (2.9%), Northern European (16.38%), Southern European (13.96%)*	1
BIHi005-A hiPSC	Asian	-	*Asian*	1
BT-20	White	European	*African (5.3%), Northern East Asian (9.54%), Northern European (43.6%), Southern European (41.56%)*	3
BT-474	White	European	*African (.6%), Northern East Asian (2.47%), Northern European (70.19%), Southern European (26.74%)*	4
BT-549	White	European	*African (.21%), Northern East Asian (3.6%), South Asian (7.62%), Northern European (26.05%), Southern European (62.78%)*	1
BV173	White	European	*African (1.38%), Northern East Asian (2.14%), South Asian (2.51%), Northern European (52.2%), Southern European (41.77%)*	2
BxPc-3	White	European	*African (1.55%), Northern East Asian (1.41%), Northern European (69.25%), Southern European (27.79%)*	3
C33a	White	European	*African (.41%), Native American (.33%), Northern East Asian (.48%), Southern East Asian (.19%), South Asian (.82%), Northern European (66.58%), Southern European (31.19%)*	1
Caco-2	White	European	*African (3.33%), Northern East Asian (4.59%), Northern European (47.35%), Southern European (44.73%)*	3
Caki-1	White	European	*African (1.09%), Native American (.91%), Northern East Asian (2.59%), Southern East Asian (.47%), South Asian (7.88%), Northern European (28.81%), Southern European (58.26%)*	2

CAL27	White	European	*African (2.22%), Northern East Asian (2.6%), (Northern European (54.66%), Southern European (40.51%)*	2
CALU3	White	European	*African (.63%), Northern East Asian (2.09%), Northern European (67.76%), Southern European (29.51%)*	2
Cal51	White	European	*Native American (.75%), Northern East Asian (.86%), South Asian (2.28%), Northern European (55.8%), Southern European (40.31%*	1
CaOV3	White	European	*African (2.29%), Native American (.12%), Northern East Asian (2.22%), South Asian (15.22%), Northern European (11.3%), Southern European (68.85%)*	1
Capan-1	White	European	*African (1.95%), Native American (.56%), Northern East Asian (4.79%), Southern East Asian (.11%), Northern European (19.06%), Southern European (66.44%)*	1
CaSki	White	European	*African (1.02%), Northern East Asian (1.86%), Northern European (68.36%), Southern European (28.76%)*	1
CHP-212	White	European	*African (2.16%, Northern East Asian (1.09%), Southern East Asian (.43%), South Asian (.86%), Northern European (70.26%), Southern European (25.19%)*	1
CL1-5	Asian	East Asian	*Chinese/Taiwan*	1
COLO 205	White	European	*Northern East Asian (2.08%), Northern European (61.39%), Southern European (36.53%)*	1
COLO668	White	European	*African (2.4%), Native American (16.55%), Northern East Asian (3.63%), South Asian (3.09%), Northern European (28.55%) Southern European (45.77%)*	1
Daoy	White	European	*African (.45%), Northern East Asian (1.54%), South Asian (1.1%), Northern European (64.36%), Southern European (32.54%)*	1
DMS79	White	European	*African (.52%), Northern East Asian (.95%), South Asian (1.45%), Northern European (66.84%), Southern European (30.24%)*	1
DU145	White	European	*African (.75%), Northern East Asian (2.66%), South Asian (.04%), Northern European (63.74%), Southern European (32.8%)*	3

FADU	Asian	Central/South Asian	*African (2.06%), Northern East Asian (11.01%) South Asian (79.51%), Southern European (7.42%)*	1
GM12878	White	European	*Race defined as White/ Northern and Western European; ethnicity defined as Utah Mormon*	2
H1299	White	European	*African (.33%), Northern East Asian (2.13%), Northern European (62.86%), Southern European (34.68%)*	4
H1975	White	European	*African (.35%), Northern East Asian (1.32%), South Asian (7.46%) Northern European (30.01%), Southern European (60.85%)*	3
H3122	White	European	*Northern East Asian (1.36%), South Asian (1.48%), Northern European (65.9%), Southern European (31.26%)*	2
H460	White	European	*Northern East Asian (1.96%), South Asian (.15%), Northern European (65.65%), Southern European (32.24%)*	3
H520	White	European	*Northern East Asian (.92%), Northern European (63.58%), Southern European (35.5%)*	1
HaCaT	White	European	*Caucasian*	2
HCC-1806	Black or African American	African	*African (80.71%), Northern East Asian (3.77%), Northern European (6.8%), Southern European (8.72%)*	2
HCC-1395	White	European	*African (2%), Northern East Asian (1.43%), South Asian (.64%), Northern European (62.97%), Southern European (32.96%)*	2
HCC-70	Black or African American	African	*African (70.85%), Northern East Asian (5.47%), Northern European (3.61%), Southern European (20.07%)*	2
HCC-827	White	European	*African (1.62%), Native American (.39%), Northern East Asian (3.47%), South Asian (7.09%), Northern European (19.48%), Southern European (67.96%)*	1
HCG-1 hiPSC	Asian	East Asian	*Japanese*	1
HCT-116	White	European	*Native American (.08%), Northern East Asian (1.16%), South Asian (.97%), Northern European (64.85%) Southern European (32.93%)*	7
HeLa	Black or African American	African	*African (64.74%), Native American (.77%), Northern East Asian (2.26%), Northern European (19.45%), Southern European (12.78%)*	28

HEp-2	Black or African American	African	*African American*	1
Hep3B	Black or African American	African	*African (71.82%), Native American (.52%), Northern East Asian (3.16%), Northern European (8.8%), Southern European (15.69%)*	1
HepG2	White	European	*African (2.83%), Native American (20.88%), Northern East Asian (4.48%), Northern European (22.82%), Southern European (48.99%)*	8
HFL-1	White	European	*Caucasian*	1
HGC-27	Asian	East Asian	*Northern East Asian (77.61%), Southern East Asian (21.46%), South Asian (.22%), Southern European (.7%)*	3
HK-2	White	European	*African (3.63%), Northern East Asian (3.68%), Northern European (62.77%), Southern European (29.92%)*	1
HL60	White	European	*African (1.15%, Native American (.42%,), Northern East Asian (.4%), Northern European (71.71%), Southern European (26.32%)*	3
HN4	White	European	*Caucasian*	1
HNSCC	Asian	East Asian	*African (.32%), Native American (1.29%), Northern East Asian (77.72%), Southern East Asian (18.87%), Northern European (1.81%)*	1
HPAC	White	European	*African (.23%), Native American (.7%), Northern East Asian (2.44%), Northern European (62.15%), Southern European (34.47%)*	1
HPB-ALL	Asian	East Asian	*Northern East Asian (78.04%), Southern East Asian (20.81%), South Asian (1.14%)*	1
HPSI0714i-nufh_3	White	European	*Caucasian, British*	1
HPSI0914i-euts_1	White	European	*Caucasian, British*	1
Hs 766T	White	European	*African (.11%), Native American (.92%), Northern East Asian (1.96%), South Asian (1.54%), Northern European (62.85%), Southern European (32.625)*	1
Hs27	Black or African American	African	*Black*	1
Hs578t	White	European	*African (.53%), Northern East Asian (1.71%), Northern European (63.73%), Southern European (34.01%)*	1

HT-3	White	European	*African (2.04%), Northern East Asian (3.34%), South Asian (1.53%), Northern European (55.35%), Southern European (37.73%)*	1
HT1080	White	European	*African (.86%)%, Native American (.35%), Northern East Asian (1.36%), Southern East Asian (.29%), Northern European (68.21%), Southern European (28.94%)*	1
HT29	White	European	*African (1.57%), Native American (1.05%), Northern East Asian (3.24%), South Asian (4.8%), Northern European (26.11%), Southern European (63.24%)*	1
Huh-6	Asian	East Asian	*African (.79), Native American (.3%) Northern East Asian (80.92), Southern East Asian (17.99%),*	1
Huh-7	Asian	East Asian	*African (1.56%), Northern East Asian (79.68%), East Asian (13.08%), Southern European (5.69%)*	2
hTERT (SRC-4000)	White	European	*Caucasian*	1
hiPSC derived from cardiomyoctes (CMC-100-010-001)	White	European	*Caucasian*	1
IGROV-1	White	European	*African (.69%), Southern East Asian (1.43%), South Asian (4.55%), Northern European (46.04%), Southern European (47.29%)*	1
IPSC (HMGUi001)	White	European	*Caucasian*	1
IPSC from Fibroblast	White	European	*White*	1
Jurkat	White	European	*African (.4%), Northern East Asian (.13%), Southern East Asian (1.21%), South Asian (1,08%), Northern European (70.73%), Southern European (26.45%)*	6
K562	White	European	*Arican (5.19%), Northern East Asian (8.4%), Northern European (43.44%), Southern European (42.97%)*	12
KMRC-20	Asian	East Asian	*African (3.3%), Northern East Asian (77.74%), Southern East Asian (14.66%), Southern European (4.3%)*	1
L02	African American	African	*African American*	1
LM7	White	European	*Caucasian*	1
LN229	White	European	*Northern East Asian (2.23%), South Asian (.02%), Northern European (59.47%), Southern European (38.27%)*	1
LNCap	White	European	*Caucasian*	1

MCF-7	White	European	*African (.74%), Northern East Asian (4.2%), Northern European (56.91%), Southern European (38.15%)*	25
MCF10A	White	European	*White*	11
MDA-MB-231	White	European	*African (1.95%), Northern East Asian (2.44%), South Asian (7.42%), Northern European (28.16%), Southern European (60.03%)*	21
MDA-MB-436	White	European	*African (1.41%), Northern East Asian (2.21%), Northern European (63.9%), Southern European (32.49%)*	2
MDA-MB-453	White	European	*Northern East Asian (2.43%), South Asian (.43%), Northern European (63.01%), Southern European (34.13%)*	3
MDA-MB-468	Black or African American	African	*African (80.3%), Northern East Asian (6.19%), Southern European (13.51%)*	5
ME-180	White	European	*African (1.51%), Native American (.16%), Northern East Asian (1.49%), Northern European (64.3%), Southern European (32.53%)*	1
MG63	White	European	*African (3.1%), Native American .69%), Northern East Asian (2.05%), South Asian (5.07%), Northern European 23.83%), Southern European (65.26%)*	2
MIA Paca-2	White	European	*African (1.15%), Northern East Asian (3.82%), South Asian (8.7%), Northern European (22.54%), Southern European (63.79%)*	2
MKN28	Asian	East Asian	*Northern East Asian (83.28%), Southern East Asian (16.71%)*	1
MKN45	Asian	East Asian	*African (1.54%), Northern East Asian (85.37%), Southern East Asian (9.42%), Southern European (3.67%)*	1
MM1.s	Black or African American	African	*African American*	1
Nalm-6	White	European	*African (.12%), Native American (.55%), Southern East Asian (2.1%), Northern European (75.01%), Southern European (22.21%*	4
NCI-H441	White	European	*African (.09%), Northern East Asian (3.45%), South Asian (1.1%), Northern European (61.67%), Southern European (33.69%)*	1

Unreported Cell-lines: 368T1, 373T1, 393T1, 393T1, 393T5, 802T1, ACGII-1 hiPSC, B721.221, BCR-ABL (+) Cell-line, C25, CAOV8, CC3 hiPSCs, CFPAC-1, COLO699, CSC14 hESCs, DPSC, EA.hy926, EBV-LCL, Ect1/E6E7, EJ, FM-6, GES-1, GSC23, GT-TdTom hESC, H2B-RFP, H9, HBE, HEK293/HEK293T/293T, HFF, hPSF (Garlick Lab), IPSC-IM90, hIPSC (Hochedlinger Lab), HMC3, HMEC-1, HN6, HONE1, HPDE6-C7, HPNE, HRVECs, HT12356, HUES9, CHANG, LMX1A-eGFP, LP9, Me275, MGC-803. MRC-5, Pa01c, Pa02c, Pa03c, Pa04c, patient-derived tumor cell-line, PEO4, Phoenix-ampho, ReNCell, RUCDi002-A hiPSC, SCC6, SeAx, SEES4 hESC, SEES5 hESC, SEES6 hESC, SEES7 hESC, SUNE1, SVG, TERT-hBMSCs, UM9, UMGi001-A hiPSC, UMGi005-A hiPSC, UWB1.289, WA H9 hESC, WA01

Supplemental Table 1. Table representing the ancestral breakdown for all cell lines that had ancestry available (n=212). This was done through three different ancestral groupings: 1) United States Censuses based ancestry (Black or African American, White, Asian, Native Hawaiian or Pacific Island, American Indian, and Two or more races), 2) genotyping studies-based ancestry (African, European, East Asian, Central and South Asian, and Mixed Ancestry), and 3) whatever was directly reported in a primary or secondary source. The table also includes the frequency (*f*) of which these cell lines appear within the database. All cell lines that had unreported ancestry were listed below the table.

**Ancestries of all Primary Samples**				
**Primary Samples**	**US Census Based Ancestry**	**Genotyping Studies Based Ancestry**	**Directly Stated in Primary or Secondary Source**	***(f)***
Human Carotid Artery Endothelial Cells	White	European	*Caucasian*	1
Human Blood	Asian	East Asian	*Chinese (from Hainan Province)*	1
Human Blood	Asian	East Asian	*Chinese*	1
Human Blood	Asian	East Asian	*Chinese*	1
Human Sputum	Asian	East Asian	*Chinese*	1
Human Umbilical Vein Endothelial Cells	White	European	*European*	1
Liver Cancer Cells	Asian	East Asian	*Chinese*	1
Macrophages	Asian	-	*Asian*	1
Peripheral Blood Mononuclear Cells	Asian	East Asian	*Han Chinese*	1
Peripheral Blood Mononuclear Cells	White	European	*Australian*	1
Peripheral Blood Mononuclear Cells	Black or African American	African	*Kenyan*	1
Peripheral Blood Mononuclear Cells	Black or African American	African	*Malian*	1
T cells	Asian	-	*Asian*	1

Supplemental Table 2. Table representing the ancestral breakdown for all primary samples that had ancestry reported (n=13). This was done through three different ancestral groupings: 1) United States Censuses based ancestry (Black or African American, White, Asian, Native Hawaiian or Pacific Island, American Indian, and Two or

more races), 2) genotyping studies-based ancestry (African, European, East Asian, Central and South Asian, and Mixed Ancestry), and 3) whatever was directly reported in a primary or secondary source. The table also includes the frequency (*f*) of which these primary samples appear within the database. All other incidences of primary samples had unreported ancestry.
